# Saudi Women's Experiences of Sexual and Relational Changes During the Menopause Transition

**DOI:** 10.1111/jan.70132

**Published:** 2025-08-07

**Authors:** Samar Alotaibi, Sharron Hinchliff, Parveen Ali

**Affiliations:** ^1^ School of Allied Health Professions, Nursing and Midwifery University of Sheffield Sheffield UK; ^2^ Maternal and Child Health Nursing Department, College of Nursing Shaqra University Shaqra Saudi Arabia; ^3^ Doncaster and Bassetlaw Teaching Hospitals Doncaster UK

**Keywords:** intimacy, menopausal transition, menopause, midlife, nurse, phenomenology, qualitative study, Saudi Arabia, sexual life

## Abstract

**Objectives:**

This study aims to understand Saudi women's experiences of sexual and relational changes during the menopause transition.

**Design:**

A qualitative, Interpretative Phenomenological Analysis study.

**Methods:**

Sixteen Saudi women aged 45–57 who had experienced natural menopause transition were purposively selected and interviewed using semi‐structured interviews between December 2022 and March 2023. The interviews were recorded and transcribed verbatim. Participants were recruited from several sites, including hospitals, gender‐segregated schools employing female staff, and social media channels. The data were analysed using Interpretative Phenomenological Analysis.

**Results:**

Three group experiential themes were identified from the data. These included ‘The intimate relationship while going through menopause’, which explores women's experiences of intimate relationships shaped by biological and hormonal changes, cultural and social expectations, and psychological influences; ‘Perceived attractiveness and self‐confidence’, which describes how physical signs of ageing impact women's body image and self‐confidence; and ‘Managing the sexual changes during the menopause transition’, which highlights varied coping strategies and attitudes toward seeking support for sexual changes during menopause.

**Conclusion:**

Healthcare systems in Saudi Arabia must provide comprehensive menopausal care and train nurses and healthcare providers to consider women's sexual difficulties from a biopsychosocial perspective. Raising Saudi women's awareness of menopausal and sexual issues, as well as mitigating society's stereotypes, is crucial for empowering them to seek help.

**Practice Implications:**

Understanding how menopausal women experience sexual and relational changes during their menopause transition is crucial for nurses, as it enables them to provide appropriate care that supports both physical and emotional well‐being. As nurses recognise these experiences, they can offer guidance, reduce stigma, and enhance women's quality of life.

**Reporting Method:**

The study adhered to the Consolidated Criteria for Reporting Qualitative Research.

**Patient or Public Contribution:**

No Patient or Public Involvement.

## 1 Introduction

Menopause is one of the physiological milestones in a woman's life. The menopausal transition refers to the shift from reproductive to non‐reproductive status and typically spans 4–10 years surrounding the final menstrual cycle (Greendale et al. 2020). The average age at onset of menopause is 46.7 years, with a range of 42–51 years (Chadha et al. 2016). The World Health Organisation estimates that over 1.2 billion women will be menopausal by 2030 (Srinivasan and Martens 2018). However, adapting to menopausal changes can be challenging due to its impact on many aspects of their physical and psychological well‐being, including their sexual well‐being (Thomas and Thurston 2016).

Perimenopause and menopause are marked by a gradual decline in sex hormones, which can have an impact on women's health and sexual well‐being. Menopausal women are more likely to experience sexual difficulties due to the interplay of personal, relational, and social influences on health (Eftekhar et al. 2016). Genitourinary syndrome and hypoactive sexual desire disorder are the most commonly studied clinical conditions during menopause (Simon et al. 2018). Both of these conditions affect other aspects of sexuality such as arousal, orgasm, and ultimately sexual satisfaction (Kingsberg et al. 2023). These sexual issues affect women's quality of life, psychological health, and interpersonal relationships. Understanding what shapes sexual well‐being during midlife helps women make sense of their experiences and enables healthcare professionals to provide meaningful guidance to those navigating related concerns (de Boer 2023). It has been argued that qualitative insights into women's sexual experiences during midlife remain underexplored and underutilised, despite their critical role in deepening understanding of how various factors influence sexual well‐being (Wellings et al. 2023). In Saudi Arabia, women's experience of menopause, including sexuality and intimate relationships, has been under‐researched despite its prevalence in Western cultures.

## 2 Background

As hormonal changes alone cannot fully explain sexual experiences during midlife and menopause, a biopsychosocial approach is required (Simon et al. 2018). Most aspects of sexuality are strongly influenced by sociocultural factors (Ussher et al. 2015). Psychosocial factors, including partner availability, attitudes toward ageing, and emotional well‐being, have been argued to be more influential in determining sexual satisfaction than hormonal fluctuations, demonstrating the importance of psychological and relational dynamics (Avis et al. 2018; Ussher et al. 2015). Research has shown that the quality of relationships and social factors such as fatigue and lack of communication have a greater impact on sexual wellbeing in midlife than age or menopause stage, underscoring the necessity for considering broader psychosocial factors (Wellings et al. 2023). The physical or psychological health of women or their partners during midlife can significantly affect their quality of life and sexual function (Harder et al. 2019). Moreover, cultural stigmas, embarrassment, and lack of knowledge of management options often make it difficult to discuss sexual health openly, leaving many women without the care they need (Nappi and Cucinella 2015), especially in conservative societies (Yang et al. 2016).

In Arabic, menopause is commonly described as “sen al yaas”, which means “the age of despair or hopelessness”. Often, menopause is associated with negative connotations, such as the loss of fertility and a decline in femininity (AlSwayied et al. 2024; Azar et al. 2016). Women's reproductive roles are often emphasised in Arab communities, and the end of menstruation may feel like a loss, compounding feelings of inadequacy. However, in the Middle East, women's construction of their sexuality has been neglected significantly (Azar et al. 2016). In Saudi Arabia, there is an increase in the number of women approaching menopause, and approximately one‐third of women will live in postmenopause (AlSwayied et al. 2024). The latest available study investigating the onset age of menopause in Saudi Arabia reported a mean age of 48.1 ± 5.9 years (Addar et al. 2005). Additionally, a recent review reveals a pooled prevalence of 47.27% for sexual problems among middle‐aged Saudi women, highlighting the significance of the issue (Fang et al. 2024). The increasing number of women approaching menopause suggests that we should try to enhance women's quality of life by understanding their health and well‐being needs.

Sexuality discussions in Saudi Arabia are reported to be taboo and socially unacceptable (Alomair et al. 2022a). The majority of women in Saudi Arabia are Muslim, and studies suggest that cultural factors play a key role in the suppression of Muslim women's discussions about sexual and reproductive health (Merghati‐Khoei et al. 2014; Shariati et al. 2014). In many Muslim countries, polygamy may reinforce patriarchal structures and contribute to gender inequalities in marriage (Asad et al. 2021). Additionally, the emphasis on modesty, while religiously grounded, is often culturally amplified in a way that limits women's access to open conversations about sexuality (Azar et al. 2021). Therefore, women are unlikely to seek support or information about menopause due to limited open discussion, compounded by the modesty surrounding reproductive and sexual health (AlSwayied et al. 2024).

Nurses and midwives play a crucial role in the assessment of sexual health and the provision of counselling and clinical services, as recognised in global health research (Fennell and Grant 2019). Understanding women's experiences of sexual function during the menopause transition is essential for nurses and healthcare providers to offer evidence‐based, patient‐centred care. It allows them to address common concerns such as relationship changes, physical discomfort, and emotional distress, which are often overlooked in clinical practice (Azar et al. 2022). Although sexual experiences during the menopause transition have been explored in various contexts (Chen et al. 2023; Vidayanti and Retnaningsih 2020; Wellings et al. 2023), there is a lack of research focusing on how cultural and societal norms influence Saudi women's experiences. The conservative nature of Saudi society makes discussing sexual health a sensitive subject, which may result in misinformation, stigma, or unmet healthcare needs (Alomair et al. 2022b). Other conservative societies face similar issues, where cultural taboos and gender norms limit women's ability to communicate openly about their sexual health during menopause (Agunbiade and Gilbert 2020; Chen et al. 2023). Understanding women's experiences through a biopsychosocial lens that acknowledges biological, psychological, and social dimensions can enrich the interpretation of the phenomenon under study. Existing literature in diverse cultural contexts has explored women's sexual experiences during menopause (Agunbiade and Gilbert 2020; Chen et al. 2023; Harder et al. 2019). However, there is limited qualitative research focusing on the lived experiences of Saudi women within their unique sociocultural context. Therefore, exploring their experiences with menopause and sexuality is essential to ensuring that their health needs are understood and adequately addressed.

## 3 The Study

This study aims to undertake an in‐depth exploration of Saudi women's experiences of sexual and relational changes during the menopause transition between the ages of 45–59 years. This study helps us understand how sociocultural factors shape menopausal sexuality, which may be applicable to other culturally similar regions. Using qualitative research methods to examine the experiences of sexual function during menopause provides rich, context‐specific insights into cultural, social, and personal factors influencing this process. This approach fills critical knowledge gaps, guiding culturally appropriate healthcare interventions and policy development.

## 4 Materials and Methods

### 4.1 Study Design

We used a qualitative study design, involving Interpretative Phenomenological Analysis (IPA) to better understand Saudi women's experiences of sexual and relational changes during the menopause transition. IPA is an approach developed to understand participant's experiences (Smith et al. 2009). This method suits this study since it focuses on understanding, “people's lived experience and how they make sense of it in the context of their personal and social worlds” (Smith and Nizza 2022, p.3). This study was conducted as part of the first author's PhD research.

### 4.2 Participants

The sample included Saudi women aged 45–59 who experienced the natural menopause transition and were willing to share their experiences voluntarily. Purposive sampling was utilised to select participants based on specific criteria, ensuring access to a particular perspective relevant to the study's focus. The study was conducted in Riyadh, the capital city of Saudi Arabia. Participants were recruited from several sites, including hospitals, gender‐segregated schools employing female staff, and social media channels such as WhatsApp. The first author approached potential participants in both settings, introduced herself and the study, and distributed flyers outlining the study's purpose and contact information. The recruitment flyer was also shared on several WhatsApp groups for women's networks. Interested participants were invited to contact the researcher for more information. Participants were informed that the first author was a PhD student with a background in nursing, conducting the study as part of her academic degree. They were made aware that the study aimed to explore women's lived experiences of menopause and that the researcher's interest in the topic was motivated by the need to explore and empower menopausal women in Saudi Arabia through giving voice to their experiences. There were no prior relationships with participants.

A total of 16 women aged 45–57 were included in the final sample, and all names used in this study were pseudonyms. Participants' demographic characteristics are presented in Table 1. Menopausal status was classified into peri‐ and post‐menopausal status. The classification is based on information provided by participants during the interview. The term perimenopausal was defined as participants who have experienced changes in their menstrual regularity and length, terminated their menstrual cycle for less than one year, and have experienced menopausal symptoms such as hot flashes, vaginal dryness, and diminished libido. Participants who have not had menstruation for more than one year are classified as postmenopausal.

**TABLE 1:** Participant demographics.

Pseudonym	Age	Marital status	Education level	Number of pregnancies and children	Menopausal status
Ahlam	46	Married	Bachelor's degree	Pregnancies: 8 Children: 6	Perimenopausal
Zainab	50	Married	High school	Pregnancies: 10 Children: 9	Perimenopausal
Albandri	47	Married	Diploma	Pregnancies: 7 Children: 7	Perimenopausal
Laila	54	Married	High school	Pregnancies: 11 Children: 5	Postmenopausal
Huda	52	Married	Diploma	Pregnancies: 6 Children: 3	Perimenopausal
Rahmah	56	Married\Separated	High school	Pregnancies: 8 Children: 8	Perimenopausal
Badriah	52	Widow	Bachelor's degree	Pregnancies: 6 Children: 6	Postmenopausal
Amira	54	Married	Bachelor's degree	Pregnancies: 5 Children: 5	Postmenopausal
Munierah	55	Married	Bachelor's degree	Pregnancies: 4 Children: 4	Perimenopausal
Mona	45	Married	Bachelor's degree	Pregnancies: 5 Children: 4	Perimenopausal
Seham	46	Married	Diploma	Pregnancies: 5 Children: 5	Perimenopausal
Wadha	57	Married	High school	Pregnancies: 4 Children: 2	Perimenopausal
Khlood	46	Married	Bachelor's degree	Pregnancies: 6 Children: 5	Perimenopausal
Haya	55	Married	Bachelor's degree	Pregnancies: 1 Children: 0	Postmenopausal
Salma	54	Married	Bachelor's degree	Pregnancies: 4 Children: 4	Postmenopausal
Hend	52	Married	Bachelor's degree	Pregnancies: 5 Children: 5	Postmenopausal

### 4.3 Data Collection

A semi‐structured interview was conducted by the first author to gain in‐depth insight into the experiences of sexual and relational changes among Saudi women. In IPA, this method was recommended as the most flexible one. The purpose of choosing this method over focus group discussions was to provide flexibility in preparing the wording of sensitive questions and to offer privacy to the participants. The first author had prior experience studying menopause knowledge among women in Saudi Arabia, which informed her understanding of the topic, and she was aware of the cultural sensitivities surrounding discussions of sexuality in Saudi Arabia, which informed both the development of the interview guide and the approach to data collection and interpretation. Additionally, she received formal training in qualitative research methods, including an extensive workshop focused on preparing for and conducting semi‐structured interviews. The first author shared the same gender, language, and cultural background as the participants. These shared characteristics were considered to support rapport‐building and encourage open dialogue. Reflexivity was maintained throughout the study process to acknowledge and manage any potential bias or assumptions. Field notes were also made during and after each interview to capture contextual observations and personal reflections.

The practical guide to conducting IPA interviews was helpful in structuring questions as open‐ended questions and providing prompts for participants to share their experiences (Smith and Nizza 2022). The interview questions were developed after reviewing the literature on this topic. Throughout developing the interview schedule, we carefully chose the words when asking about the phenomenon. For example, we know the term ‘hopeless age’ is widely used in Saudi Arabia when talking about menopause. However, we use the term menopause instead, which in Arabic means the cessation of menstruation. The interviews began with participants sharing their sociodemographic details, thoughts, and emotions about menopause, and as trust and comfort grew, the conversation gradually shifted toward exploring their sexual experiences. Participants were invited to reflect on their experiences of menopause and midlife by responding to open‐ended questions such as: “Tell me about your menopause, how has that been for you?” and “How do you describe your feelings approaching menopause?” Once rapport was established, more specific questions were asked regarding bodily changes, sexual experiences, and communication with partners and others. Prompts included changes in physical symptoms such as hot flashes, sleep disturbance, and vaginal dryness, as well as shifts in body image and the ability to discuss sexual concerns. Participants were also asked whether they had spoken with anyone, such as a spouse, friend, or healthcare professional, about these changes and how those conversations were received.

The interviews were conducted either face‐to‐face or via telephone, based on the participants' preferences. The interviews took place between December 2022–March 2023. Each participant was assured of strict confidentiality and invited to select a convenient time and private location for the face‐to‐face or telephone interview, which was conducted in a private room at either the women's school or the hospital. The interviews duration varied, with a maximum of one and a half hours and a minimum of twenty minutes. Thirteen potential participants did not progress to the interview, with reasons including concerns about having their voices recorded, lack of response to follow‐up communication, and personal time constraints and responsibilities. No repeat interviews were conducted.

### 4.4 Ethical Considerations

Ethics approval was obtained from the University of Sheffield (approval number 048747). An Information Sheet and Consent Form were provided for potential participants. The information sheet contains a description of the study's purpose, the topic of the interview, possible risks and benefits, and how data would be collected. Furthermore, there is information about who might be able to access the recordings, as well as invitations to view the transcripts and/or publications. Transcripts of the interviews were anonymised to ensure their confidentiality. After transcription, the recordings were erased.

### 4.5 Data Analysis

All audio‐recorded interviews were transcribed by the first author to get familiar with the data. The analysis process follows Smith's IPA steps (Smith et al. 2022). First, reading and re‐reading involved deep immersion in the data through multiple close, line‐by‐line readings of transcripts and listening to audio recordings. Second, exploratory noting was conducted directly on hard copies with wide margins, enabling descriptive comments on content, linguistic observations of specific word choices, and conceptual insights at a more interpretative level. Third, constructing experiential statements involved distilling these notes into concise summaries that capture the core experiential meanings articulated by participants. Fourth, naming personal experiential themes required identifying connections among experiential statements and labelling each theme to reflect its essence. Fifth, moving to the next case, each transcript was analysed independently to allow novel themes to emerge, consistent with IPA's idiographic commitment. Finally, developing group experiential themes involved synthesising patterns across all cases to highlight both unique and shared elements of women's menopausal experiences (Smith et al. 2022). An example of the data analysis process is provided in Table 2.

**TABLE 2:** Example of the data analysis.

Group experiential themes (GETs)	Experiential statement	Personal experiential themes	Quote excerpt
The intimate relationship while going through menopause	The absence of her husband's appreciation and his offensive remarks caused her to lose sexual desire.	Lack of partner support in menopause	‘My husband used to make me feel like I'm a Waaw woman. Now, no, he sometimes makes me hear words that might indicate that he is not cooperating with me because this is the menopause period. So, I hear from him such words as you have grown up and grown older, not as you used to be. So, I have no desire to put pressure on myself or to act because of him’ (Hend, 52 years)
Perceived attractiveness and self‐confidence	Her diminished confidence and shame over her sagging skin created a barrier to physical closeness, making her retreat from being seen by her husband.	Embodied insecurities in menopausal transition	‘I never used to feel shy about my body. But now, now, I feel shy about my body. I don't let him see it because there are sagging places in my body, so you feel shy, I mean. So, it is difficult’ (Haya, 55 years)
Managing the sexual changes during the menopause transition	She dismisses the idea of seeking medical help for her low sexual desire, anticipating a judgmental response that blames her age and offers no meaningful support.	Dismissal of medical consultation	‘I felt that it was not worth that much… Also, if I go to the doctor during this period, he will tell me that you are over fifty, for sure, the desire will decrease, so he will give me clear excuses. So, it is unnecessary’ (Salma, 54 years)

### 4.6 Rigour and Reflexivity

Data collection and analysis were part of the PhD studies of the first author. The first author interviewed all the women in Arabic and then transcribed the interviews. This study followed the criteria for trustworthiness proposed by Guba and Lincoln (1994) to ensure rigour in the research process. Credibility was maintained through iterative questioning, prolonged engagement, and continuous reflexivity. The field notes assisted the interpretative process by providing contextual depth and reflexive awareness. Participants were asked various questions about their sexual and relational change experiences during their menopausal transition, enabling an in‐depth exploration of the topic. Data and findings were contextualised and detailed to ensure transferability. Documenting each methodological decision throughout the research process ensured dependability. Additionally, all authors contributed to reviewing the developing themes, and consensus was reached through regular discussions of the data interpretation to ensure coherence and accountability. Confirmability was maintained by including rich data extracts in the Findings section and ongoing reflexivity. The richness of the data was ensured using in‐depth, semi‐structured interviews that allowed participants to share detailed and personal accounts of their experiences. This approach is consistent with the aims of IPA, which prioritises depth over breadth. Rather than seeking data saturation, the focus in IPA was on generating rich, interpretative insights through close engagement with each individual transcript and iterative analysis (Hale et al. 2008). Transcripts were not returned to participants, as transcript review was not part of the study design. In keeping with the interpretative phenomenological approach, participant meaning was explored through in‐depth analysis and reflexive interpretation. Member checking was instead achieved through discussions among the research team and consensus on the developing themes.

## 5 Findings

The group experiential themes discussed below are ‘The intimate relationship while going through menopause’ and ‘Perceived attractiveness and self‐confidence’ and ‘Managing the sexual changes during the menopause transition’. Each theme has subthemes, which highlight the similarities and differences in participants' experiences.

### 5.1 The Intimate Relationship While Going Through Menopause

Women's experiences of intimacy during the menopause transition are explored in this theme, illustrating the diversity of experiences around intimate relationships that the participants experienced.

#### 5.1.1 Intimacy in the Context of Marriage

When talking about the sexual changes the participants experienced while going through menopause, many noted the biological changes such as vaginal dryness and lack of sexual desire. Half of the sample mentioned experiencing vaginal dryness whether due to menopause or their health conditions. Their experience of vaginal dryness affected their intimate relationship and caused intense pain, emphasising the interconnectedness of biological factors with the relational aspects of their menopause experience:

The dryness, since the start of menopause. But this year, this year, from one year to now, it is the strongest, the strongest period of dryness is now… I feel it after intercourse… And I become tired, for example, and it lasts a whole day, and I feel pain in the area. (Hend, 52 years, postmenopausal)

Participants also cited their health conditions when discussing vaginal dryness, especially diabetes, which can reduce vaginal lubrication due to hormonal and vascular changes:

I am a diabetic, so I used to take care of the area. Of course, it became dry, but what I noticed was that the area became dry for sometimes and sometimes not I mean, I found that it bothered me with my husband when it happened. (Amira, 54 years, postmenopausal)

Many women have reported decreasing sexual desire after menopause, describing a change in their interest in intimacy. They often compare it with their level of desire in the past while expressing less motivation to engage in sexual activity, stating that their interest in sex had diminished since reaching menopause. This highlights the interaction between biological changes, such as hormonal shifts and relational factors:

I no longer have sexual desire, it is not like before… the desire was like any girl who would love to have intercourse, to be with her husband when he asks for her. Now, I hope he doesn't ask for it (Laughter). (Salma, 54 years, postmenopausal)

The lack of education about sexual health and well‐being in their earlier lives was reported as a reason for their decreasing sexual desire. This lack of desire makes participants feel guilty about the lack of sexual education in their family's upbringing and their focus on superficial matters instead of the foundation of the marital relationship, underscoring the complex interplay between early life experiences, social norms, and individual psychological responses to sexual relationships:

It already does not exist (the sexual desire). It was zero from the beginning. How about now?… I don't know, I don't know. Maybe this always focuses on my being a bit negligent in these matters. Unfortunately, I don't know. Maybe because our families don't teach us to be aware of many things. They teach us how to cook, listen, clean, and sweep. They don't teach us about that thing, which is unfortunately, unfortunately, the basic criterion in evaluating a woman. If she is good or not good? (Munierah, 55 years, perimenopausal)

Participants also discussed how their partner's health issues affected their sexual lives during the menopause transition, providing insight into how relationship dynamics are central to intimacy and sexual well‐being at this time. One participant described how her partner's diabetes led to erectile dysfunction and the loss of their intimate relationship when asked about changes in sexual desire:

There has been nothing between me and my husband for almost 13 years, nothing…the problem is with my husband… he has diabetes, and after that, he couldn't do anything. (Zainab, 50 years, perimenopausal)

However, not all participants expressed having a lack of sexual desire. Some participants stated having a sexual desire as they go through menopause, especially for those who had difficult life circumstances, such as marriage at a young age or being separated from their husbands for a long time. They expressed their desire to marry after going through menopause to live a normal and satisfying sexual relationship:

I mean, it exists, I mean this deprivation. I mean, it still exists. I mean, it is definitely true that the desire decreases. However, I see that I mean, I still have the ability and competency that I can remarry and live for a period with a husband, I mean, for example, a normal relationship. (Badriah‐ 52 years, postmenopausal)

The findings demonstrate that the participants' sexual desire was impacted by their partner's role in supporting their wives during the menopause transition, illustrating the interplay of psychological and social factors. Having a partner who does not understand or want to discuss the menopausal symptoms and bodily changes caused participants to face difficulty adjusting to the situation, resulting in the loss of their sexual desire:

My husband used to make me feel like I'm a Waaw woman. Now, no, he sometimes makes me hear words that might indicate that he is not cooperating with me because this is the menopause period. So, I hear from him such words as you have grown up and grown older, not as you used to be. So, I have no desire to put pressure on myself or to act because of him. (Hend, 52 years, postmenopausal)

However, some participants found that their partners' support and understanding of their menopausal experience and the changes that they came through were helpful and contributed to a positive intimacy experience:

He sees me as a woman who satisfies him from looking for other women… He spoils me, and this thing actually reflects on my mood, I also become active with him… and this affects my mood so much, and I feel I'm an attractive woman, you know. (Ahlam, 46 years, perimenopausal)

#### 5.1.2 The Importance of Being Committed to Sexual Activities

The findings demonstrate that the participants placed a high value on sexual activity in their marital relationships. There was a common belief among them that a sexual relationship is necessary for the fulfilment of religious duties and marital satisfaction. Indeed, they expressed their fear of the angel's curse if they refused. An angel's curse is a spiritual punishment rather than a physical penalty. It reflects divine disapproval and a deprivation of blessings. It is widely known that an angel's curse is invoked when a wife refuses her husband's request for sexual relations without a valid reason:

As for me, I'm doing a duty only, and we don't want to upset the husband, or as they said, we won't be cursed by an angel. (Hend, 52 years, postmenopausal)

Besides that, accepting the sexual relationship is a religious duty and is deeply ingrained in the participants' culture, it was also imperative to keep the husband within the scope of the relationship. In this context, patriarchal values have a profound impact on gender roles, relationships, and sexuality, where women are expected to priorities marriage stability and the satisfaction of their husbands over their own autonomy:

It is a marital duty. But if I'm, if, if I don't give him what he wants, who will give it to him? You know! Do I need to let him head toward what is forbidden, for example, or he marry again, for example? So, yeah either I let him marry again or I meet this need for him. This is my view. (Haya, 55 years, postmenopausal)

However, some of the participants expressed a sense of blame for their partners. They admitted that they neglected an important right of their partner. This was especially in the context where their husbands experienced increased sexual desire, affecting their quality of life:

Honestly, he has been affected by this (the lack of intimacy)… he no longer leads his normal life normally… It is fine for me that he seeks someone else… Because I didn't give him his right (sexual right), I admitted. (Khlood, 46 years, perimenopause)

### 5.2 Perceived Attractiveness and Self‐Confidence

The participants described how their perceptions of attractiveness influence their self‐confidence and intimate relationships during the menopause transition and midlife. The changes to physical appearance that come with age can evoke complex emotional responses, affecting how women view themselves. This theme explores how bodily changes affect the women's feelings of attractiveness during their menopausal transition, reflecting how psychological factors play a key role in experiences of menopause.

#### 5.2.1 Emotional Responses to Ageing and Bodily Changes

The physical changes experienced by participants during the menopause transition included an increase in weight, loose skin, and grey hair. These changes impacted how they expressed their attractiveness and intimate relationships, particularly in a society that places a value on beauty and youth. Participants expressed their dissatisfaction with being able to gain weight faster than previously and were concerned about how others would perceive their attractiveness at this age:

I'm gaining weight faster!! before, I wasn't like that… honestly, it is for sure that I'm upset!! Upset because I always have a perfect body and people appreciate my perfect body and like that, so that's why I'm upset. (Ahlam, 46 years, perimenopausal)

Participants expressed that grey hair was a constant reminder of ageing, something they did not want to acknowledge or accept due to the negative stereotype around menopause and ageing. It demonstrates how these women's self‐image is challenged by physical changes, perceptions of ageing, and their desire to maintain a youthful appearance:

There is a lot of this (the grey hair) that what makes me feel bored. I dyed it. I just dyed it yesterday (Laughs). It bothers me a lot… So when I see it, I became annoyed and felt that my skin was pale and tired and that I had grown old. (Seham, 46 years, perimenopausal)

However, some participants showed resistance to societal beauty standards and revealed their acceptance of bodily changes, including weight gain. Their spiritual beliefs served as a source of reassurance, enabling them to stand against societal expectations:

I accept myself, even if those (other people) say that I need to do gastric sleeve surgery. I feel that God created me like this. I accept myself as my Lord created me, so I am reconciled. (Amira, 54 years, postmenopausal)

#### 5.2.2 The Importance of the Partner's View of Body Changes

The feelings toward bodily changes were intensified within the context of their intimate relationship and how their husbands would perceive them. Participants became self‐conscious about their body's shape and wondered if their husband would accept it:

It is embarrassing (the sagging), I mean, it is impossible to wear something, for example, so short, or wearing shorts between my friends. Even with my husband, by God, it's very embarrassing, but I don't know if my husband has become accustomed to me or if he is not prone to hurting me. God knows, I don't know. (Hend, 52 years, postmenopausal)

The participants exhibited sensitivity to their bodily changes during the menopause transition, affecting their sexual responses. This represents the societal standard of beauty as young and fit, which makes them anticipate judgement even if their partner has not expressed negative views. They conveyed feelings of embarrassment about their bodily changes, which led them to avoid exposing their bodies to their husbands:

I never used to feel shy about my body. But now, now, I feel shy about my body. I don't let him see it because there are sagging places in my body, so you feel shy, I mean. So, it is difficult. (Haya, 55 years, postmenopausal)

The participants expressed their emotional needs for validation and reassurance about their body image during the menopause transition. Indeed, they felt gratified when their husbands made positive comments about their bodies, which increased their sexual confidence. This emphasises the importance of the partner's appreciation of their appearance:

He is happy with my body, thank God. Even when I say I want to do a diet and I want to take some care of my body, he says to me, ‘No, no, don't change. The opposite is true, It's so wonderful and nice’. (Salma, 54 years, postmenopausal)

### 5.3 Managing the Sexual Changes During the Menopause Transition

Throughout this theme, the experiences and perspectives of participants on ways to cope with sexual changes at menopause transition were varied. There was a difference in attitudes between participants who sought help and those who did not. There were a variety of sources of help, ranging from healthcare professionals to social assistance from family and friends.

#### 5.3.1 Seeking Help for the Sexual Changes

Various participants discussed how they sought assistance with reproductive and sexual changes, whether medical or preferring not to get help. Many participants complained about vaginal dryness and differed in the way they sought help for it. For some, the medical consultation for dyspareunia was unhelpful:

I spoke to a doctor. I don't know. Maybe the doctor I consulted was not good. I don't know…the thing that it really differs, I feel it differs, it is just a pain in itself. Previously, I wasn't in pain at the time of the relationship not like now, there is a little bit of pain, and I think that also prevents the desire itself. I discussed this matter with the doctor, and she said ‘No, there is nothing wrong with it’ (Haya, 55 years, postmenopausal)

Participants rely heavily on shyness when it comes to seeking help for sexual issues. This indicates the taboo around talking about sexual issues and seeking help in their community. Several participants reported that they had difficulty seeking help for sexual issues due to a lack of confidence. In response to the question of whether they had considered using lubricants, some participants said:

No, no, no, never… I don't know. I don't like it, I don't know. I have shame in these things, and I don't reveal it. I keep silent. (Wadha, 57 years, perimenopausal)

Some participants thought it was unnecessary to discuss sexual issues. The reasons were related to their earlier expectations about the response of the healthcare providers and their unrelatedness to health. They consider it unnecessary and expect the doctor to explain that this is normal with ageing:

I felt that it was not worth that much… Also, if I go to the doctor during this period, he will tell me that you are over fifty, for sure, the desire will decrease, so he will give me clear excuses. So, it is unnecessary. (Salma, 54 years, postmenopausal)

Due to the importance of intimacy, participants expressed how difficult it was for them to accept a sexual relationship while experiencing a lack of sexual desire, resulting in many of them faking it. Indeed, they explained the difficulty of informing their husbands about this change, which forced them to fake their sexual desire and avoid open discussion:

I no longer have sexual desire, not like before. This is what I didn't tell my husband about, of course (laughter). I told him about menopause, but I didn't tell him about this…By God, it's tiring for me because I have to fake it… And it's tiring to me because I'm afraid of getting caught (laughing). (Salma, 54 years, postmenopausal)

However, the experience of going through menopause and sexual changes offered some participants the agency to refuse sexual relationships, which was not possible in their earlier lives. They described how they were able to refuse sexual relationships by creating physical or mental excuses:

Previously, I haven't dared to say no, for example. But now, I have the nerve to say no…You may say, for example, that I am tired, I am physically tired, or I am mentally tried to excuse yourself… Previously, you tolerated it, you knew it was normal. But now I can't stand it anymore. It's over. (Haya, 55 years, postmenopausal)

#### 5.3.2 Looking for Possible Approaches

Several approaches were mentioned by participants to cope with menopausal sexual changes, whether medically, spiritually, or socially. Participants mentioned the need for a solution to the vaginal dryness issue. Even though they suffered from severe vaginal dryness, they chose to use non‐recommended methods, such as olive oil, because they found it to be effective:

The doctor told me that (It was fine), meaning that one of the glands was completely closed and completely malfunctioning… I mean, she was the one who said that natural olives are good, no harm, and so I used it. (Badriah, 52 years, postmenopausal)

Additionally, some mentioned that their doctor suggested using prophetic medicine, a traditional treatment, as a good solution to menopausal symptoms, including vaginal dryness:

She (her doctor) didn't give me anything. I remember I asked her about hijama (Cupping) and she said “Yes, hijama is good, this is suggested by the Messenger”. (Hend, 52 years, postmenopausal)

However, some participants received appropriate support from their healthcare providers. Their sexual issues were handled in a way that provided an assurance, and they were able to adapt to these issues:

I have been following up with my obstetrics and gynaecology doctor since the day of the bleeding. She prepared me. I mean, she gave me creams… I didn't feel it was a problem. (Amira, 54 years, postmenopausal)

However, some participants preferred their own traditional method to prevent vaginal dryness. They have a strong belief that feminine‐enhancing drinks are important for them because they protect against vaginal dryness. This represents how cultural beliefs and traditional practices influence women's approaches to managing sexual health issues during menopause:

A bottle of anise, fennel seeds, and fenugreek tea is what I drink every day. Only, so, these things make women maintain moisture may God bless and protect you. It let the hormones be active. (Rahmah, 56 years, perimenopausal)

Cosmetic surgeries in the vaginal area have been mentioned by many participants for tightening the area or for improving the colour. Most of them, however, indicated that they did not have enough information about it. They mentioned their colleagues' positive experiences in using these methods such as Laser Vaginal Rejuvenation (LVR). Some of them were reluctant to perform any cosmetic procedures on their vaginal area, as they were afraid that the result may be negative:

I heard about it (vaginal cosmetic surgery). But I won't do it. However, I see my friends and so on. The level of engagement in it has skyrocketed. LVR, and enlargement to the area and things that I don't know. I do not support that… the result may be the opposite. And you have a man and like this, and it is not nice that he even knows that you did something like that or something, by God, I feel that it is not nice. (Salma, 54 years, perimenopausal)

Most participants did not mention Hormonal Replacement Therapy (HRT) when they looked for a treatment for sexual changes and other symptoms during the menopause transition. They often did not have enough knowledge about it. They expressed a strong desire to learn about HRT and tried to seek medical advice for that but did not find it helpful in some cases, as they wanted to know why it is not recommended:

I asked her about these oestrogen hormone pills, and she said, ‘No, no, I can't give you anything now. (Badriah, 52 years, postmenopausal)

The lack of information about HRT led some participants to seek other women's experiences as a guide to deciding whether or not to use HRT. Sharing other experiences was common in the participants' accounts and considered to be a valid source of information in many cases:

Oestrogen is the first thing to take… Yah, but I don't want anything to harm me. I want something right. I mean, I will see people who tried it, the honest people, not commercials. Those who tried and worked for them, I want to see myself. (Mona, 45 years, perimenopausal)

## 6 Discussion

This study is among the limited research exploring women's sexual and relational changes while going through the menopause transition in Saudi Arabia, adding to the gap in the existing knowledge. The findings reveal that women's sexual and relational experiences during menopause were shaped by an interplay of biological, psychological, and social factors. They emphasised the importance of intimate relationships for marital and religious purposes, and how a lack of them could negatively impact a woman's quality of life. Participants reported diverse menopause experiences and described various approaches to managing sexual changes. These approaches included actively seeking medical consultations, feeling hesitant due to the taboo surrounding discussions about sexuality, seeking insights from others' experiences, or adhering to traditional practices.

Although the findings indicate that many participants experienced a lack of sexual desire after menopause, which is consistent with previous studies (Hinchliff et al. 2018; Javadivala et al. 2018; Thomas et al. 2017), the lack of sexual health education in their adolescent life and the taboo around these topics were reported as an important reason for their lack of sexual desire in both their earlier life and during the menopause transition. In a similar vein, one study found that Lebanese participants with lower levels of education tended to disapprove of women expressing interest in sexuality during midlife (Azar et al. 2016). As that study was conducted within Arabic and primarily Muslim culture, it has similarities to the present study and reflects how a lack of sexual health education could impact women's lives. This suggests that nurses and healthcare providers in Saudi Arabia and other Muslim countries need to be trained to educate women about menopause, sexual health, and overall health. They also need to communicate sensitively and respectfully to address misconceptions and cultural taboos around menopause and sexual health and well‐being and empower women to seek help.

The sexual lives of the participants were influenced by interpersonal dynamics. Participants cited their partner's health and sexual issues as a reason for their declining sexual activity, indicating the important role that interpersonal relationships play in understanding how women experience menopause and intimate relationships. This supports existing literature when women reported a decline in their sexual activities due to their partner's health or sexual issues (DeLamater et al. 2019; Ussher et al. 2015). These findings contextualise menopausal women's sexual experiences within the context of their interpersonal and social contexts, which need to be taken into account by nurses and healthcare providers during the assessment of menopausal women's experiences. Additionally, engaging women's partners in the discussions and educating them about menopause changes can strengthen relationship intimacy and enhance support.

The participants in this study described their physical changes, such as weight gain, sagging, and grey hair, during the menopausal transition as a reason for losing their attractiveness. This is in line with previous literature where women expressed their concern about ageing and bodily change signs (Amini and McCormack 2019; Moghasemi et al. 2018), where a sense of attractiveness is an important indicator of the level of sexual desire among women (Thomas et al. 2021). Similarly, a recent Saudi study revealed that women expressed concerns about losing their attractiveness during menopause, using terms such as “old‐fashioned” and “becoming expired,” which led some to hide their menopausal status from their partners (AlSwayied et al. 2024). This highlights the impact of the negative stereotypes associated with ageing and the perceived loss of desirability upon women's self‐perceptions. Additionally, norms around body size begin to shift due to Western influences, leading non‐Westerners to adopt Western ideals, including the desire for thinness (Swami 2015). As suggested by Melisse et al. (2022), the thin ideal may increasingly influence body dissatisfaction in Saudi Arabia. Consequently, women may feel self‐conscious about their body changes during midlife, feeling under pressure to fulfil these imposed standards. Cultural context must be considered when understanding the participants' concerns about weight gain and body changes during menopause. Understanding the psychological and cultural factors that influence women's sexual desire during menopause is crucial for nurses and healthcare providers as they can help menopausal women embrace their body changes with confidence by encouraging positive body image and self‐acceptance. They need to promote self‐care strategies, such as exercise, mindfulness, and relaxation techniques, in order to improve self‐esteem and overall well‐being.

The importance of intimate relationships was described clearly among Saudi women in their menopausal transition. Quality of life, in particular the quality of marriage, depends on the quality of sexual life for many participants. Despite the taboo around sexual topics in Saudi culture, these findings highlight its importance for women in their menopausal transition. This importance puts a high value on sexual relationships for the sake of the marriage, which is stated clearly in the previous literature (DeLamater et al. 2019; Thomas et al. 2018). However, this importance increases when it comes to the role of women toward their husbands in Islam. Similarly, menopausal women in Islamic countries like Iran and Lebanon reported the importance of satisfying their partners' sexual needs, linking that to their Islamic teachings. The sexual difficulties that women encounter in their menopausal transition lead them to fake their sexual desire just to satisfy their husbands; these responses have been mentioned in many cultures (Amini and McCormack 2021; Ghazanfarpour et al. 2018), reflecting the gender role differences. Nurses and healthcare providers can understand the importance of sexual aspects and provide a safe, non‐judgmental space for women to express their concerns and explore the impact of suppressing their needs. In addition, they can offer them advice on healthy communication strategies and refer them to a counsellor or sex therapist if needed.

Our findings highlighted the different ways menopausal women chose to deal with the sexual changes. Following traditional beliefs was common among the participants to deal with vaginal dryness. Indeed, none of the participants in this study reported using any type of HRT; instead, they reported a lack of knowledge about it. Unlike previous studies, which found that women use HRT to alleviate vaginal dryness and enhance sexual desire (Harder et al. 2019; Yang et al. 2016), there is a general lack of discussion in Saudi society about menopause and its management options, which reflects the limited knowledge participants displayed about HRT. The finding is supported by a recent Saudi study, which found that more than half of the participants lacked adequate knowledge about HRT (Basri et al. 2024). The results indicate that cultural, educational, and healthcare factors may contribute to women's poor understanding of menopausal treatment options. Therefore, women can be educated on HRT by nurses and healthcare providers to provide accurate information about its benefits, risks, and suitability for managing sexual changes. They should also allocate time for women to discuss sexual issues, clarify misconceptions, answer questions, and discuss alternatives to ensure women make informed decisions.

### 6.1 Strengths and Limitations of the Work and Recommendations for Further Research

To our knowledge, this is the first in‐depth qualitative study of women's lived experiences of sexual and relational changes during the menopause transition in Saudi Arabia. Most previous menopause research in Saudi Arabia has employed quantitative methods, relying on descriptive cross‐sectional study designs (Abdel‐Salam et al. 2021; Al‐Musa et al. 2017; AlQuaiz et al. 2017; Elazim et al. 2014). Therefore, this qualitative study provides new insights that could influence both policy and healthcare practices in the Saudi context, bringing attention to an aspect of women's health that is often overlooked. To ensure a deeper understanding of Saudi women's experiences of sexual and relational changes during the menopause transition, the interviews were conducted in Arabic using local dialects. We recruited participants to represent diverse socioeconomic backgrounds, including a variety of marital, educational, and menopausal statuses. Future research should build on these findings by conducting more qualitative studies in other Middle Eastern or Gulf countries to explore cultural influences on menopause and sexuality in depth. Additionally, mixed‐methods research could provide a more comprehensive understanding by integrating both qualitative insights and quantitative data on women's experiences of menopause, sexual experience, and healthcare needs.

We attempted to recruit women from different places; however, most were recruited from women's schools. This may have given working women more opportunities to participate than non‐working women, which may limit the transferability of the findings to the wider Saudi female population. Despite the importance of having the male partner's perspectives and experiences about menopause and intimate changes, this study did not include them due to the sensitivity of the topic as well as the nature of the conservative culture in Saudi Arabia. However, future research should include Saudi men's perspectives on menopause and intimate relationships.

Although menopause and sexuality are sensitive topics, women were willing to openly discuss them in this study. These conversations highlight the importance of the subject and suggest that, while menopause and sexuality remain taboo, more open conversations are needed in research and healthcare.

### 6.2 Practice Implications

Nurses should understand women's experiences of menopause and sexual changes from a biopsychosocial lens, not just from a biological standpoint. This holistic approach can provide a more complete picture of how women experience menopause and sexuality, including the cultural and religious influences. Taking part in training in menopause care and understanding psychosocial impacts such as sexual issues and stigma would be useful for continuing professional development. Menopause‐related sexual issues need to be addressed in a sensitive, respectful, and neutral manner, acknowledging concerns such as reduced libido, dry vaginal tissue, and emotional impact on intimate relationships. Nurses play an important role in supporting women with these concerns and encouraging them to seek help for sexual problems.

## 7 Conclusion

This study sheds light on a hidden and culturally sensitive topic in Saudi society that has been largely unexplored in previous research. It highlights the diversity of Saudi women's experiences of sexual and relational changes during the menopause transition, underscoring the importance of addressing these experiences through a biopsychosocial lens by nurses and healthcare providers, as they play a crucial role in enhancing the care of menopausal women in the Saudi healthcare system. Nurses and healthcare professionals who work in women's health can help to empower women by fostering their confidence, encouraging open conversations about menopause and sexual difficulties, and helping them overcome the silence often surrounding these issues. Additionally, they can work to dispel negative stereotypes about menopause and ageing by providing accurate, accessible information to raise awareness.

## Author Contributions

A.S., H.S., A.P. made substantial contributions to conception and design, or acquisition of data, or analysis and interpretation of data; involved in drafting the manuscript or revising it critically for important intellectual content; given final approval of the version to be published. Each author has participated sufficiently in the work to take public responsibility for appropriate portions of the content; agreed to be accountable for all aspects of the work in ensuring that questions related to the accuracy or integrity of any part of the work are appropriately investigated and resolved.

## Ethics Statement

Ethics approval was obtained from the University of Sheffield Ethics Committee (approval number 048747). All research procedures adhered to the ethical guidelines for conducting research involving human participants.

## Consent

Informed consent was obtained from all participants before their involvement in the study. Participants were provided with detailed information about the study's purpose, their rights, and the voluntary nature of participation.

## Conflicts of Interest

The authors declare no conflicts of interest.

## Supporting information


**Data S1:** jan70132‐sup‐0001‐Supinfo.pdf.

## Data Availability

The data that support the findings of this study are available on request from the corresponding author. The data are not publicly available due to privacy or ethical restrictions.

## References

[jan70132-bib-0001] Abdel‐Salam, D. M. , R. A. Mohamed , R. R. Alruwaili , F. S. Alhablani , R. M. Aldaghmi , and R. E. Alghassab . 2021. “Postmenopausal Symptoms and Their Correlates Among Saudi Women Attending Different Primary Health Centers.” International Journal of Environmental Research and Public Health 18, no. 13: 6831. 10.3390/ijerph18136831. 34202185 PMC8296999

[jan70132-bib-0002] Addar, M. , M. El Desouki , and Z. Babay . 2005. “Correlates of Age at Menopause and Osteoporosis in Saudi Women.” Clinical and Experimental Obstetrics & Gynecology 32, no. 2: 135–137. 16108401

[jan70132-bib-0003] Agunbiade, O. M. , and L. Gilbert . 2020. “The Night Comes Early for a Woman: Menopause and Sexual Activities Among Urban Older Yoruba Men and Women in Ibadan, Nigeria.” Journal of Women & Aging 32, no. 5: 491–516. 10.1080/08952841.2019.1593772. 30922211

[jan70132-bib-0004] Al‐Musa, H. M. , R. A. Ahmed , A. S. Alsamghan , et al. 2017. “The Prevalence of Symptoms Experienced During Menopause, Influence of Socio‐Demographic Variables on Symptoms and Quality of Life Among Women at Abha, Saudi Arabia.” Biomedical Research‐India 28, no. 6: 2587–2595.

[jan70132-bib-0005] Alomair, N. , S. Alageel , N. Davies , and J. V. Bailey . 2022a. “Barriers to Sexual and Reproductive Wellbeing Among Saudi Women: A Qualitative Study.” Sexuality Research & Social Policy 19, no. 3: 860–869. 10.1007/s13178-021-00616-4.

[jan70132-bib-0006] Alomair, N. , S. Alageel , N. Davies , and J. V. Bailey . 2022b. “Sexual and Reproductive Health Knowledge, Perceptions and Experiences of Women in Saudi Arabia: A Qualitative Study.” Ethnicity & Health 27, no. 6: 1310–1328. 10.1080/13557858.2021.1873251. 33554633

[jan70132-bib-0007] AlQuaiz, A. M. , A. Kazi , F. Habib , M. AlBugami , and A. AlDughaither . 2017. “Factors Associated With Different Symptom Domains Among Postmenopausal Saudi Women in Riyadh, Saudi Arabia.” Menopause 24, no. 12: 1392–1401. 10.1097/gme.0000000000000931. 28697042

[jan70132-bib-0008] AlSwayied, G. , R. Frost , and F. L. Hamilton . 2024. “Menopause Knowledge, Attitudes and Experiences of Women in Saudi Arabia: A Qualitative Study.” BMC Women's Health 24, no. 1: 624. 10.1186/s12905-024-03456-7. 39581992 PMC11587664

[jan70132-bib-0009] Amini, E. , and M. McCormack . 2019. “Medicalization, Menopausal Time and Narratives of Loss: Iranian Muslim Women Negotiating Gender, Sexuality and Menopause in Tehran and Karaj.” Womens Studies International Forum 76: 102277. 10.1016/j.wsif.2019.102277.

[jan70132-bib-0010] Amini, E. , and M. McCormack . 2021. “Older Iranian Muslim Women's Experiences of Sex and Sexuality: A Biographical Approach.” British Journal of Sociology 72, no. 2: 300–314. 10.1111/1468-4446.12805. 33341961 PMC8246973

[jan70132-bib-0011] Asad, N. , R. Somani , N. Peerwani , et al. 2021. ““I Am Not the Person I Used to Be”: Perceptions and Experiences of Menopausal Women Living in Karachi, Pakistan.” Post Reproductive Health 27, no. 4: 199–207. 34806468 10.1177/20533691211060099

[jan70132-bib-0012] Avis, N. E. , X. Zhao , C. B. Johannes , M. Ory , S. Brockwell , and G. A. Greendale . 2018. “Correlates of Sexual Function Among Multi‐Ethnic Middle‐Aged Women: Results From the Study of Women's Health Across the Nation (SWAN).” Menopause 25, no. 11: 1244–1255. 10.1097/gme.0000000000001226. 30358720

[jan70132-bib-0013] Azar, M. , N. Azar , T. Kroll , and C. Bradbury‐Jones . 2021. “Should I Seek Help for Sexual Difficulties? Middle‐Aged Lebanese Women's Views.” Journal of Sex & Marital Therapy 47, no. 7: 635–655. 10.1080/0092623x.2021.1934208. 34154514

[jan70132-bib-0014] Azar, M. , T. Kroll , and C. Bradbury‐Jones . 2016. “Lebanese Women and Sexuality: A Qualitative Inquiry.” Sexual & Reproductive Healthcare 8: 13–18. 10.1016/j.srhc.2016.01.001. 27179372

[jan70132-bib-0015] Azar, M. , T. Kroll , H. Chakhtoura , V. Gebran , and S. D. Sailian . 2022. “Nurses and Midwives Role in Patient Sexual Health Assessment: A Cross‐Sectional Study.” Sexuality and Disability 40, no. 3: 583–598. 10.1007/s11195-022-09739-x.

[jan70132-bib-0016] Basri, T. H. , N. M. Alharbi , R. S. Almohammed , et al. 2024. “Women's Knowledge, Attitude, and Practice Toward Menopause and Hormone Replacement Therapy in Saudi Arabia.” International Journal of Medicine in Developing Countries 8, no. 4: 1702.

[jan70132-bib-0017] Chadha, N. , V. Chadha , S. Ross , and B. C. Sydora . 2016. “Experience of Menopause in Aboriginal Women: A Systematic Review.” Climacteric 19, no. 1: 17–26. 10.3109/13697137.2015.1119112. 26653073

[jan70132-bib-0018] Chen, J. , H. Zhai , H. Jin , X. Li , P. Zhang , and R. Chen . 2023. “Sexual Experiences of Postmenopausal Women in China: A Qualitative Study.” Sexual Medicine 11, no. 6: qfad062. 10.1093/sexmed/qfad062. 38058407 PMC10696166

[jan70132-bib-0019] de Boer, M. 2023. ““Becumming” Oneself as One Relates to Others: An Empirical Phenomenological Study About Sexual Identity Work in Menopause.” Sexualities 28: 450–469. 10.1177/13634607231200969.

[jan70132-bib-0020] DeLamater, J. , E. R. Koepsel , and T. Johnson . 2019. “Changes, Changes? Women's Experience of Sexuality in Later Life.” Sexual and Relationship Therapy 34, no. 2: 211–227. 10.1080/14681994.2017.1412419.

[jan70132-bib-0021] Eftekhar, T. , M. Dashti , M. Shariat , F. Haghollahi , F. Raisi , and A. Ghahghaei‐Nezamabadi . 2016. “Female Sexual Function During the Menopausal Transition in a Group of Iranian Women.” Journal of Family & Reproductive Health 10, no. 2: 52–58. 27648093 PMC5026668

[jan70132-bib-0022] Elazim, H. A. , S. M. Lamadah , and L. G. Al Zamil . 2014. “Quality of Life Among of Menopausal Women in Saudi Arabia.” Jordan Medical Journal 48, no. 4: 227–242. 10.12816/0025073.

[jan70132-bib-0023] Fang, Y. , F. Liu , X. Zhang , et al. 2024. “Mapping Global Prevalence of Menopausal Symptoms Among Middle‐Aged Women: A Systematic Review and Meta‐Analysis.” BMC Public Health 24, no. 1: 1767. 10.1186/s12889-024-19280-5. 38956480 PMC11220992

[jan70132-bib-0024] Fennell, R. , and B. Grant . 2019. “Discussing Sexuality in Health Care: A Systematic Review.” Journal of Clinical Nursing 28, no. 17–18: 3065–3076. 10.1111/jocn.14900. 31017326

[jan70132-bib-0025] Ghazanfarpour, M. , T. Khadivzadeh , and R. L. Roudsari . 2018. “Sexual Disharmony in Menopausal Women and Their Husband: A Qualitative Study of Reasons, Strategies, and Ramifications.” Journal of Menopausal Medicine 24, no. 1: 41–49. 10.6118/jmm.2018.24.1.41. 29765926 PMC5949307

[jan70132-bib-0026] Greendale, G. A. , A. S. Karlamangla , and P. M. Maki . 2020. “The Menopause Transition and Cognition.” JAMA 323, no. 15: 1495–1496. 10.1001/jama.2020.1757. 32163094

[jan70132-bib-0027] Guba, E. G. , and Y. S. Lincoln . 1994. “Competing Paradigms in Qualitative Research.” In Handbook of Qualitative Research, edited by N. K. Denzin and Y. S. Lincoln , 105–117. Sage Publications, Inc.

[jan70132-bib-0028] Hale, E. D. , G. J. Treharne , and G. D. Kitas . 2008. “Qualitative Methodologies II: A Brief Guide to Applying Interpretative Phenomenological Analysis in Musculoskeletal Care.” Musculoskeletal Care 6, no. 2: 86–96. 10.1002/msc.113. 17628038

[jan70132-bib-0029] Harder, H. , R. M. L. Starkings , L. J. Fallowfield , et al. 2019. “Sexual Functioning in 4,418 Postmenopausal Women Participating in UKCTOCS: A Qualitative Free‐Text Analysis.” Menopause‐The Journal of the North American Menopause Society 26, no. 10: 1100–1109. 10.1097/gme.0000000000001377. 31290761 PMC6791508

[jan70132-bib-0030] Hinchliff, S. , J. Tetley , D. Lee , and J. Nazroo . 2018. “Older Adults' Experiences of Sexual Difficulties: Qualitative Findings From the English Longitudinal Study on Ageing (ELSA).” Journal of Sex Research 55, no. 2: 152–163. 10.1080/00224499.2016.1269308. 28139143

[jan70132-bib-0031] Javadivala, Z. , E. Merghati‐Khoei , C. Underwood , M. Mirghafourvand , and H. Allahverdipour . 2018. “Sexual Motivations During the Menopausal Transition Among Iranian Women: A Qualitative Inquiry.” BMC Women's Health 18: 191. 10.1186/s12905-018-0684-z. 30470219 PMC6260987

[jan70132-bib-0032] Kingsberg, S. A. , B. Adler , J. Metropoulos , and S. S. Faubion . 2023. “The Yin and Yang of GSM and Low Sexual Desire.” Climacteric 26, no. 4: 323–328. 10.1080/13697137.2023.2194529. 37083058

[jan70132-bib-0033] Melisse, B. , E. F. van Furth , and E. de Beurs . 2022. “The Saudi‐Arabic Adaptation of the Body Shape Questionnaire (BSQ34): Psychometrics and Norms of the Full Version and the Short Version (BSQ8C).” Frontiers in Psychology 13: 1046075. 10.3389/fpsyg.2022.1046075. 36532987 PMC9754054

[jan70132-bib-0034] Merghati‐Khoei, E. , F. Sheikhan , N. Shamsalizadeh , H. Haghani , Y. R. Yousofnia Pasha , and T. Killeen . 2014. “Menopause Negatively Impacts Sexual Lives of Middle‐Aged Iranian Women: A Cross‐Sectional Study.” Journal of Sex & Marital Therapy 40, no. 6: 552–560. 10.1080/0092623x.2013.796577. 24308863

[jan70132-bib-0035] Moghasemi, S. P. , G. P. Ozgoli , F. P. Ahmadi , and M. P. Simbar . 2018. “Sexual Experience of Iranian Women in Their Middle Life: A Qualitative Approach.” International Journal of Community Based Nursing and Midwifery 6, no. 1: 47–55. 29344535 PMC5747572

[jan70132-bib-0036] Nappi, R. E. , and L. Cucinella . 2015. “Advances in Pharmacotherapy for Treating Female Sexual Dysfunction.” Expert Opinion on Pharmacotherapy 16, no. 6: 875–887. 10.1517/14656566.2015.1020791. 25732267

[jan70132-bib-0037] Shariati, M. , R. Babazadeh , S. A. Mousavi , and K. M. Najmabadi . 2014. “Iranian Adolescent Girls' Barriers in Accessing Sexual and Reproductive Health Information and Services: A Qualitative Study.” Journal of Family Planning and Reproductive Health Care 40, no. 4: 270–275. 10.1136/jfprhc-2013-100856. 25183530

[jan70132-bib-0038] Simon, J. A. , S. R. Davis , S. E. Althof , et al. 2018. “Sexual Well‐Being After Menopause: An International Menopause Society White Paper.” Climacteric 21, no. 5: 415–427. 10.1080/13697137.2018.1482647. 29987939

[jan70132-bib-0039] Smith, J. A. , P. Flowers , and M. Larkin . 2009. Interpretative Phenomenological Analysis: Theory, Method and Research. SAGE Publications.

[jan70132-bib-0040] Smith, J. A. , P. Flowers , and M. Larkin . 2022. Interpretative Phenomenological Analysis: Theory, Method and Research. 2nd ed. SAGE.

[jan70132-bib-0041] Smith, J. A. , and I. E. Nizza . 2022. Essentials of Interpretative Phenomenological Analysis. American Psychological Association.

[jan70132-bib-0042] Srinivasan, V. , and M. G. Martens . 2018. “Hormone Therapy in Menopausal Women With Fibroids: Is It Safe?” Menopause 25, no. 8: 930–936. 10.1097/gme.0000000000001105. 29613885

[jan70132-bib-0043] Swami, V. 2015. “Cultural Influences on Body Size Ideals.” European Psychologist 20, no. 1: 44–51. 10.1027/1016-9040/a000150.

[jan70132-bib-0044] Thomas, H. M. , M. Hamm , T. Krishnamurti , R. Hess , S. Borrero , and R. C. Thurston . 2021. ““How Much Desire Should I Have?”: A Qualitative Study of Low Libido in Postmenopausal Women.” Journal of Women & Aging 34: 649–657. 10.1080/08952841.2021.1977070. 34543166 PMC8934312

[jan70132-bib-0045] Thomas, H. N. , M. Hamm , R. Hess , S. Borrero , and R. C. Thurston . 2017. “Patient‐Centered Outcomes and Treatment Preferences Regarding Sexual Problems: A Qualitative Study Among Midlife Women.” Journal of Sexual Medicine 14, no. 8: 1011–1017. 10.1016/j.jsxm.2017.05.014. 28647404 PMC5538956

[jan70132-bib-0046] Thomas, H. N. , M. Hamm , R. Hess , and R. C. Thurston . 2018. “Changes in Sexual Function Among Midlife Women: “I'm Older… and I'm Wiser”.” Menopause 25, no. 3: 286–292. 10.1097/GME.0000000000000988. 29088016 PMC5821528

[jan70132-bib-0047] Thomas, H. N. , and R. C. Thurston . 2016. “A Biopsychosocial Approach to Women's Sexual Function and Dysfunction at Midlife: A Narrative Review.” Maturitas 87: 49–60. 10.1016/j.maturitas.2016.02.009. 27013288 PMC4808247

[jan70132-bib-0048] Ussher, J. M. , J. Perz , and C. Parton . 2015. “Sex and the Menopausal Woman: A Critical Review and Analysis.” Feminism & Psychology 25, no. 4: 449–468. 10.1177/0959353515579735.

[jan70132-bib-0049] Vidayanti, V. , and L. N. Retnaningsih . 2020. “Sexual Experience Among Postmenopausal Women in Yogyakarta: A Qualitative Study.” Bali Medical Journal 9, no. 1: 80–85. 10.15562/bmj.v9i1.1627.

[jan70132-bib-0050] Wellings, K. , L. Gibson , R. Lewis , J. Datta , W. Macdowall , and K. Mitchell . 2023. ““We're Just Tired”: Influences on Sexual Activity Among Male‐Partnered Women in Midlife; A Mixed Method Study.” Journal of Sex Research 60, no. 9: 1304–1317. 10.1080/00224499.2023.2165613. 36757684 PMC7619711

[jan70132-bib-0051] Yang, C.‐F. , N. J. Kenney , T.‐C. Chang , and S.‐R. Chang . 2016. “Sex Life and Role Identity in Taiwanese Women During Menopause: A Qualitative Study.” Journal of Advanced Nursing 72, no. 4: 770–781. 10.1111/jan.12866. 26708447

